# The Possible Role of MOPr-DOPr Heteromers and Its Regulatory Protein RTP4 at Sensory Neurons in Relation to Pain Perception

**DOI:** 10.3389/fncel.2020.609362

**Published:** 2020-11-13

**Authors:** Wakako Fujita

**Affiliations:** Department of Medical Pharmacology, Nagasaki University Graduate School of Biomedical Sciences, Nagasaki, Japan

**Keywords:** opioid, RTP4, pain, DRG, heteromer

## Abstract

Heteromers between mu opioid receptor (MOPr) and delta opioid receptor (DOPr) (i.e., MOPr-DOPr heteromer) have been found to be expressed in different brain regions, in the spinal cord, and in dorsal root ganglia. Recent studies on this heteromer reveal its important pathophysiological function in pain regulation including neuropathic pain; this suggests a role as a novel therapeutic target in chronic pain management. In addition, receptor transporter protein 4 (RTP4) has been shown to be involved in the intracellular maturation of the MOPr-DOPr heteromers. RTP4 appears to have unique distribution *in vivo* being highly expressed in sensory neurons and also macrophages; the latter are effector cells of the innate immune system that phagocytose foreign substances and secrete both pro-inflammatory and antimicrobial mediators; this suggests a possible contribution of RTP4 to neuronal immune-related pathological conditions such as neuropathic pain. Although RTP4 could be considered as an important therapeutic target in the management of pain via MOPr-DOPr heteromer, a few reports have supported this. This review will summarize the possible role or functions of the MOPr-DOPr heteromer and its regulatory molecule RTP4 in pain modulation at sensory neurons.

## Introduction

In the nervous system including central and peripheral neurons, G-protein coupled receptors (GPCRs) play important roles in the signal transduction by neurotransmitters. For example, opioid receptors are widely distributed along the pain pathways and play a role in pain attenuation. Over the last decade studies have shown that heteromerization of GPCRs alters their pharmacological characteristics or their function (Gomes et al., 2016). That is, heteromers exhibit novel pharmacology, such as altered ligand-binding properties, G-protein coupling, and trafficking, that differs from that of homomers. Several groups including ours have focused on the physiological role of heteromerization between mu opioid receptors (MOPr) and delta opioid receptors (DOPr) and found that it may be involved in mechanisms modulating antinociceptive tolerance to morphine (He et al., 2011; Fujita et al., 2019). Thus early studies showed that DOPr antagonists decreased tolerance to the analgesic effects of morphine, a MOPr agonist (Abdelhamid and Takemori, 1991; Schiller, 2010), increased morphine-mediated antinociception (Gomes et al., 2004), and animals lacking DOPr or the gene-encoding preprotachykinin A do not exhibit morphine tolerance (Zhu et al., 1999; Guan et al., 2005). This was followed by studies by several groups that detected the presence of MOPr-DOPr heteromers and characterized their properties [reviewed in Rozenfeld and Devi (2011), Gomes et al. (2016), Ugur et al. (2018), Zhang et al. (2020)]. One of the studies showed that the MOPr-DOPr heteromer is constitutively associated with beta-arrestin, and treatment with DAMGO alone (a MOPr agonist) leads to sustained MAPK phosphorylation (characteristic of beta-arrestin-mediated phosphorylation), while a combination of DAMGO and TIPPψ (a DOPr antagonist) leads to a rapid and transient increase (characteristic of G protein-mediated signaling) as seen in cells expressing only MOPr (Rozenfeld and Devi, 2007). This signaling switch from G-protein (for MOPr) to beta-arrestin (for MOPr-DOPr heteromer) results in the cytoplasmic retention of MAPK and differential activation of downstream transcription factors (Rozenfeld and Devi, 2007). Interestingly, mice lacking beta-arrestin2 exhibit increased and prolonged morphine-induced antinociception (Bohn et al., 1999) and do not develop the antinociceptive tolerance to morphine (Bohn et al., 2000). The constitutive association of the MOPr-DOPr heteromer with beta-arrestin together with observations that chronic morphine exposure upregulates MOPr-DOPr heteromers in brain regions involved in pain signaling (e.g., rostroventral medulla and nucleus accumbens) (Gupta et al., 2010). Pierre et al. suggest the possibility that these heteromers play a pivotal role in the regulation of pain and antinociceptive tolerance by showing the co-localization of MOPr and DOPr after chronic morphine treatment (Pierre et al., 2019). Ligands targeting the MOPr-DOPr heteromer have been reported. These include bivalent ligand that contains MOPr agonist and DOPr antagonist pharmacophores separated by a spacer arm (MDAN series) and a small molecule ligand, CYM51010, that exhibit potent antinociception with reduced antinociceptive tolerance or dependence comparing to morphine (Daniels et al., 2005; Gomes et al., 2013; Fujita, 2020). Together these studies suggest that the MOPr-DOPr heteromer could be of therapeutic significance as a target for pain management.

Although not much is known about the mechanisms that regulate the biosynthesis and maturation of MOPr-DOPr heteromers, a receptor chaperone protein called receptor transporter protein 4 (RTP4), appears to play a key role in endogenous MOPr-DOPr heteromerization and in increasing MOPr-DOPr levels following long-term MOPr stimulation (Fujita et al., 2019). Furthermore, only 4 publications have reported the RTP4 interaction with GPCRs, among which 2 with MOPr-DOPr heteromer (Behrens et al., 2006; Decaillot et al., 2008; Mainland and Matsunami, 2012; Fujita et al., 2019). In these reports, as described above, the role of RTP4 as an endogenous chaperone molecule in regulation of MOPr-DOPr heteromers under physiological condition or under chronic morphine treatment has been clearly demonstrated (Decaillot et al., 2008; Fujita et al., 2019). Other 2 publications reported the role of RTP4 in regulation of taste receptors (Behrens et al., 2006; Mainland and Matsunami, 2012). They speculate that binding of RTP4 could expose an existing targeting motif within the receptor or mask an intracellular retention signal, while a detailed study on the mechanism by which the plasma membrane localization of taste receptors is regulated and on the exact roles of RTP4 will be necessary to understand how the function of receptors is achieved *in vivo* (Behrens et al., 2006). Furthermore, besides serving as a chaperone protein for the transport of GPCRs (Behrens et al., 2006; Decaillot et al., 2008; Gifford et al., 2008), RTP4 has been shown to have other physiological roles. That is, RTP4 seems to be an important marker in various cancers, is involved in hypertension and regulated by interferon as described and discussed by Fujita et al. (2019).

Interestingly, recent studies reveal that RTP4 expression is induced upon immune stimulation *in vitro* (Schoggins et al., 2011; Hoyo-Becerra et al., 2015; Nair et al., 2017; Dang et al., 2018); this suggests an effect on neuronal immune-related pathological states including neuropathic pain. In this review, we outline the possible role of the MOPr-DOPr heteromer and RTP4 in pain with an emphasis on neuropathic pain.

## The Distribution of MOPr-DOPr Heteromer in the Central and Peripheral Neurons – The Possible Implications to the Inhibition of Pain Perception

By using a selective antibody targeting the MOPr-DOPr heteromer, Gupta et al. (2010) revealed its expression in several brain regions including cortex, hippocampus, hypothalamus, nucleus accumbens, prefrontal cortex, pons, striatum, and ventral tegmental area (Gupta et al., 2010). In addition, double-knockin animals expressing MOPr fused to the red fluorescent mcherry protein (MOPr-mcherry) and DOPr fused to the green fluorescent protein (DOPr-eGFP), Erbs et al. (2015) showed that both receptors are co-expressed in selective brain regions including the lateral hypothalamus and the rostroventral medulla, the main nucleus of the trapezoid body, the hippocampus, and the pons in agreement with the findings by Gupta et al. (2010). Moreover, studies with the double-knockin mice show that dependence to morphine leads to alterations in MOPr-DOPr heteromer expression in brain regions (Pierre et al., 2019). Interestingly, co-localization of MOPr-mcherry and DOPr-eGFP fluorescent signals were observed in GABAergic interneurons that control the firing rate of glutamatergic neurons; this suggests an involvement of MOPr-DOPr heteromers in the regulation of pain. This is supported by the co-expression of the two receptors in the high throughput nociceptive pathways that include the rostral ventral medulla, lateral parabrachial nucleus, spinal cord, and dorsal root ganglion (DRG) neurons, the pseudo-bipolar neurons, with a peripheral branch that innervates their target organ and a central branch that carries the somatosensory information to the spinal cord (Erbs et al., 2015). *In situ* hybridization, single-cell PCR, electrophysiology, and immunostaining studies also provide evidence for the coexistence of functional DOPr and MOPr in a subpopulation of small-diameter peptidergic DRG neurons (Wang et al., 2010; Guerrero-Alba et al., 2018). Finally, a pharmacologic study provided evidence that MOPr and DOPr colocalize on a functionally important population of TrkA positive peptidergic nociceptors (Joseph and Levine, 2010).

Recently studies have started to elucidate the therapeutic potential for MOPr-DOPr heteromers. A study by Fujita et al. (2014) suggested that MOPr-DOPr heteromer in enteric neurons as a therapeutic target for the treatment of diarrhea-predominant irritable bowel syndrome (Fujita et al., 2014). The study found that eluxadoline, an orally active mixed MOPr agonist DOPr antagonist, exhibits an anti-diarrheal effect, and *in vitro* studies revealed that the signaling profile of eluxadoline that can be partly blocked by MOPr-DOPr heteromer-selective antibodies. Moreover, the study reported that eluxadoline can block castor oil-induced diarrhea in wild type mice and this is attenuated in DOPr knockout (DOPr^–/–^) mice indicating the involvement of DOPr probably through MOPr-DOPr heteromerization in the *in vivo* effects of eluxadoline (Fujita et al., 2014). This suggests that the actions of eluxadoline could, at least in part, be due to targeting of MOPr-DOPr heteromers in the gut. Another study by Tiwari et al. (2020) suggested that MOPr-DOPr heteromer as a potential therapeutic target for the treatment of neuropathic pain. The authors observed significant upregulation of MOPr-DOPr heteromer expression in uninjured L4 DRG neurons after L5 spinal nerve ligation (SNL) in rats. Furthermore, they found that the MOPr-DOPr heteromer targeting ligand, CYM51010, significantly inhibited mechanical and heat hypersensitivity in this pain model (Tiwari et al., 2020). These results suggest that the spinal nerve injury increases MOPr-DOPr heteromer levels in uninjured DRG neurons, and that the heteromer may be a potential therapeutic target for relieving neuropathic pain in sensory neurons. However, further studies are needed to characterize in detail the contribution of MOPr-DOPr heteromer to the modulation of pain perception.

## The Role of Receptor Transporter Protein 4 (RTP4) in the Regulation of MOPr-DOPr Heteromerization

**FIGURE 1:**
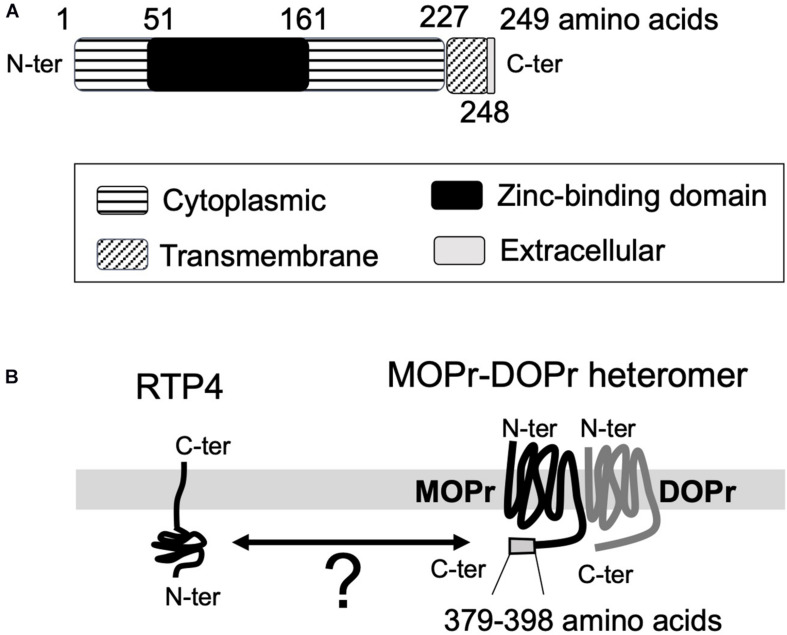
The topological information for RTP4 **(A)** and the schematic interaction between RTP4 and MOPr-DOPr heteromers **(B)**. The data was obtained via UniProt data base (uniprot.org). RTP4 (249 amino acids) has a single predicted transmembrane domain (228th to 248th amino acids) located near the C-terminal end. It is considered that the N-terminal end is intracellular (1st to 227th amino acids) and the C-terminal end (249th amino acid) is extracellular, with no signal peptide (UniProt,https://www.uniprot.org/uniprot/Q9ER80) **(A)**. Decaillot et al. (2008) have suggested that the interaction between RTP4 and MOPr-DOPr heteromer occurs in the cytoplasmic region since the region within the MOPr cytoplasmic C-terminal 29 amino acid residues (i.e., 370–398 aa) was necessary for interaction with RTP4 **(B)**.

The function and the maturation of GPCRs (and their heteromers) is known to be affected and regulated by receptor chaperone proteins (Ritter and Hall, 2009; Williams and Devi, 2010). Since the receptors must be properly folded after translation and, in most cases, transported to the plasma membrane to achieve functional activity, chaperones facilitating their transport significantly affect receptor function. Early studies with mammalian odorant receptors or taste receptors showed that receptor transporter protein family members (i.e., REEPs/RTPs) enhance receptor expression at the plasma membrane (Saito et al., 2004; Ritter and Hall, 2009). The analysis of RTP expression in mouse brain (Allen Brain Atlas)^1^ reveals that RTP4 is the most abundant subtype of the RTP family (RTP1-4) in the brain. RTP4 is known to promote cell-surface expression of a group of GPCRs involved in mediation of pain relief, bitter taste sense, or sense of smell (Saito et al., 2004; Behrens et al., 2006; Decaillot et al., 2008). RTP4 (249 amino acids) has a single transmembrane domain (228th to 248th amino acids) located near the C-terminal end. The N-terminal end is considered to be intracellular (1st to 227th amino acids) and the C-terminal end (249th amino acid) extracellular with no signal peptide (UniProt)^2^ (Figure 1A).

**FIGURE 2:**
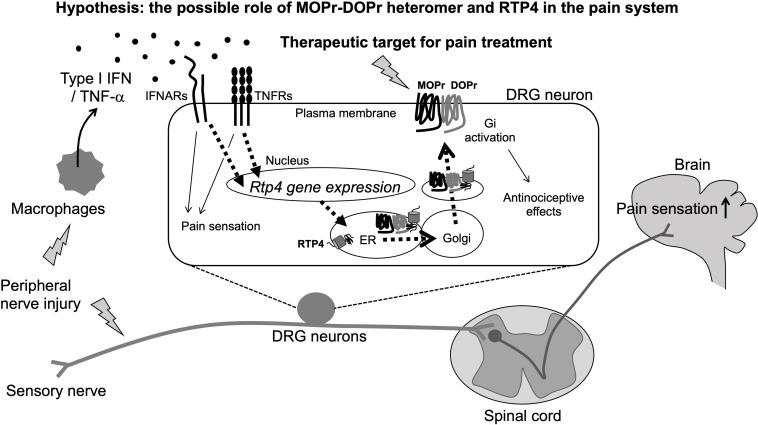
Schematic illustration of pain sensitization and the possible involvement of MOPr-DOPr heteromers and RTP4 in the regulation of pain sensation (Hypothesis). The peripheral nerve injury will stimulate peripheral immune response cells like macrophages that can produce type I IFN. RTP4 could be upregulated following the IFNARs/TNFRs activation in DRG neurons and thus induce the upregulation of MOPr-DOPr heteromer at the cell surface membrane (as a part of compensatory mechanisms occurring during neuropathic pain). Although the critical role for RTP4 and the MOPr-DOPr heteromer in pain sensitization at the spinal cord and the central nervous system is still not clear, the MOPr-DOPr heteromer and RTP4 could be a potent therapeutic target for pain treatment.

In the case of MOPr-DOPr heteromers, studies reported the requirement of RTP4 for efficient cell surface expression (Decaillot et al., 2008; Fujita et al., 2019) since in its absence a significant portion of the heteromer localized to the Golgi apparatus without being transported to the cell surface. On the other hand, the heterologous expression of RTP4 enhanced cell surface MOPr-DOPr heteromer localization (Decaillot et al., 2008); this was due to protection of the heteromer from ubiquitination and proteasomal degradation during folding and maturation. Although the details have not as yet been elucidated, the interaction between RTP4 and the MOPr-DOPr heteromer occurs via the cytoplasmic region of MOPr since cytoplasmic C-terminal 29 amino acid residues (i.e., 370–398 amino acids) of this receptor were necessary for interaction with RTP4 (Decaillot et al., 2008; Figure 1B). Interestingly, long-term MOPr stimulation by DAMGO enhanced the cell surface MOPr-DOPr heteromer expression; this was reversed by RTP4 siRNA (Fujita et al., 2019). More importantly, long-term treatment with DAMGO upregulates RTP4 levels. Upregulation in RTP4 levels and MOPr-DOPr heteromers is also seen in mice hypothalamus following chronic treatment with morphine that leads to antinociceptive tolerance (Fujita et al., 2019). While these studies show an important role for RTP4 in regulating cell surface expression of the MOPr-DOPr heteromer (Figure 2), it is still not clear if this chaperone remains associated with the heteromer at the plasma membrane and whether it modulates ligand binding to and signaling by the heteromer.

## Possible Physiological Role of RTP4 in Pain Perception – The Involvement of Cytokine Mediated RTP4 Upregulation and MOPr-DOPr Heteromer Formation in Sensory Neurons

The database of the mouse brain^3^ provides important information about RTP4 gene expression in various types of neurons (including central and peripheral) and non-neuronal cells. Taking into account the excitatory neurons and inhibitory neurons in brain regions including cortex, hippocampus, midbrain, hindbrain, thalamus, hypothalamus, telencephalon, amygdala, pallidum, septal nucleus, and spinal cord, RTP4 is highly expressed in DRG neurons (both peptidergic and non-peptidergic), sympathetic (cholinergic and noradrenergic), enteric (nitrergic and cholinergic), and noradrenergic muscle neurons. Interestingly, a Gene Expression Omnibus (GEO)^4^ DataSets revealed an increase in RTP4 gene expression in L4 and L5 DRG neurons (both contralateral and ipsilateral) 28 days post-surgery in a rat model of L5 SNL neuropathic pain. This suggests a possible physiological role for RTP4 as a regulatory molecule for pain perception at sensory neurons, since other study also reported an increase of MOPr-DOPr heteromer formation at the cell surface of the DRG neurons in neuropathic pain animals (Tiwari et al., 2020; Figure 2). Thus, upregulation of RTP4 and MOPr-DOPr heteromer levels may be a part of compensatory mechanisms occurring during neuropathic pain. More experimental studies are needed to confirm this and well as the mechanisms involved in this process.

A possible mechanism for the upregulation of RTP4 in DRG neurons during neuropathic pain could involve “interferon (IFN).” This is because the RTP4 gene can be upregulated by type I IFN (IFN-α, IFN-β, IFN -ε, IFN-κ, IFN-ω, IFN-δ, IFN-ζ, and IFN-τ) leading to RTP4 being referred to as “IFN stimulated gene” (Schoggins et al., 2011; Hoyo-Becerra et al., 2015; Nair et al., 2017; Dang et al., 2018). Peripheral immune responding cell such as plasmacytoid dendritic cells or macrophages and the central glial cells like astrocytes or microglia have been identified as main producers of type I IFNs after viral infection, and the IFNs have been shown to affect neuronal survival, neurite outgrowth leading to modulation of the glutamatergic neurotransmission under some pathological conditions including multiple sclerosis and Alzheimer’s disease (reviewed in Blank and Prinz, 2017). Interestingly, type I IFN has been reported as one of the mediators of pain (Barragan-Iglesias et al., 2020; Figure 2). Type I IFN can activate their specific receptors (IFNRs, i.e., IFNAR1, IFNAR2) resulting in downstream activation of cellular signaling and a variety of physiological responses including pain regulation. Thus, Barragan-Iglesias et al. (2020) found that type I IFNs such as IFN-α and IFN-β will act directly on nociceptors in DRG neurons, where IFNARs are expressed, to activate JAK/STAT signaling pathways leading to increased phosphorylation of mitogen activated protein kinase and of eukaryotic initiation factor 4E to promote pain hypersensitivity. They hypothesized that this mechanism may be involved in the development of pain evoked by viral infections such as HIV or herpes virus. Also, TNF-α could be one of the additional regulatory molecules of RTP4 at the periphery since it has been reported to mediate the upregulation of RTP4 expression as a neuroinflammatory signals (Intlekofer et al., 2019). As it is well known, macrophages can release TNF-α (Roy et al., 1998; Eisenstein, 2019), thus it is speculated that several inflammatory cytokines including IFN and TNF-α will be released by peripheral immune responding cells and thus may lead to upregulation of the RTP4 in sensory neuronal cells. Since these cytokines positively regulate the development of neuropathic pain (Leung and Cahill, 2010), the upregulation of RTP4 and thus increase of MOPr-DOPr heteromer levels may be a part of compensatory mechanisms occurring during neuropathic pain as described above.

In the context of pain, the immune system and the activation of peripheral macrophages or of central microglia, of resident macrophage cells of the central nervous system, have been closely related to the development of neuropathic or inflammatory pain (Kiguchi et al., 2017; Xu et al., 2020). At the injured tissue, macrophage cells or microglia will be activated leading to induction of a variety of pro-inflammatory factors that directly or indirectly sensitize pain-processing neurons in the spinal dorsal horn, DRG, and in brain regions (e.g., cortex, thalamus, amygdala, and hypothalamus) in various models of neuropathic pain including nerve damage, diabetes, and chemotherapy models (Kiguchi et al., 2017; Xu et al., 2020). According to the database Bio GPS^5^, the gene expression of RTP4 is highly detected in macrophages compared to neurons indicating a possible physiological role of RTP4 in regulation of immune responses and the development of pain, although a direct role of RTP4 in the activation or accumulation of macrophages or microglia in tissues should be investigated.

Although RTP4 was initially characterized as a GPCR chaperone protein as described above (Saito et al., 2004; Behrens et al., 2006; Mainland and Matsunami, 2012), more recent publications clearly revealed its significant role in regulation of immune responses such as IFN-related antiviral immunity (Schoggins et al., 2011; Nair et al., 2017; Dang et al., 2018; Zarei Ghobadi et al., 2019; He et al., 2020). Thus, it is suggested that RTP4 could be induced by type I IFNs and work as a negative regulator of interferons pathways. Moreover, a recent paper demonstrated that RTP4 knockout (RTP4^–/–^) mice showed a higher production of type I IFN (He et al., 2020), suggesting an important role of RTP4 in positive regulation of the immune responses, which could be explored for disease treatment and management. Taken together, RTP4 is thought to be involved in either negative or positive regulation of IFN responses, and thus it is possible that RTP4 could be involved in the pain regulatory system.

## Summary and Perspective

In this review, we summarized and hypothesized the important role of MOPr-DOPr heteromers in pain perception that is associated with neuropathic pain. Further, we suggest a possible role for RTP4, a chaperone protein of MOPr-DOPr heteromers, in pain. Although more studies are needed that show a direct contribution of MOPr-DOPr heteromers and RTP4 in neuropathic pain models, their important roles were suggested by several findings as follows.

1. MORr-DOPr heteromer and RTP4 appear to be found in DRG sensory neurons. 2. MOPr-DOPr heteromer is upregulated in the uninjured DRG by nerve injury. 3. RTP4 is a positive regulator of MOPr-DOPr heteromers in neuronal cells. 4. In DRG neurons, RTP4 can be upregulated by the nerve injury at the contralateral and the ipsilateral sites. 5. RTP4 has been shown to be upregulated by type I IFNs or TNF-α. 5. RTP4 is highly expressed in immune-related cells like macrophages that are known to produce cytokines including type I IFNs or TNF-α and to contribute to the development of neuropathic pain or pain sensation.

From these findings, we speculate that RTP4 may be a key molecule in the development of pain via modulating of MOPr-DOPr heteromer expression in neurons and thus this could be a potent therapeutic target for pain treatment. Although, the role of RTP4 in macrophages or microglia is not clear, it could contribute to the neuronal pain perception by activating the macrophages or microglia. Thus RTP4 and MOPr-DOPr heteromer are important novel therapeutic targets for the next generation pain therapeutics.

## Author Contributions

The author confirms being the sole contributor of this work and has approved it for publication.

## Conflict of Interest

The author declares that the research was conducted in the absence of any commercial or financial relationships that could be construed as a potential conflict of interest.

## Footnotes

1 https://mouse.brain-map.org/experiment/show/68444738

2 https://www.uniprot.org/uniprot/Q9ER80

3 http://mousebrain.org/genesearch.html

4 https://www.ncbi.nlm.nih.gov/geo/query/acc.cgi?acc=GSM63118

5 http://biogps.org/#goto=welcome
